# Pan-cancer analysis of TMED2: unraveling potential immune characteristics and prognostic value in cancer therapy

**DOI:** 10.3389/fimmu.2025.1578627

**Published:** 2025-05-30

**Authors:** Zhuangzhi Wang, Changning Sun, Pengfei Wang, Shouyang Lin, Xiao Wu, Yuchao Gu

**Affiliations:** ^1^ Qingdao Center of Technology Innovation for Shark Antibody Development, College of Biological Engineering, Qingdao University of Science and Technology, Qingdao, China; ^2^ School of Medicine and Pharmacy, Ocean University of China, Qingdao, China; ^3^ Department of Respiratory and Critical Care Medicine, Qingdao Central Hospital, University of Health and Rehabilitation Sciences, Qingdao, China

**Keywords:** pan-cancer analysis, immunotherapy, tumor microenvironment, protein transport, TMEDs

## Abstract

**Background:**

Transmembrane emp24 domain-containing protein 2 (TMED2) is involved in the sorting and transport of proteins between the Golgi apparatus and the endoplasmic reticulum. Recent research has identified a close association between TMED2 and tumorigenesis, yet its regulatory role and underlying mechanisms in pan-cancer signaling pathways remain unexplored.

**Methods:**

We conducted a comprehensive pan-cancer analysis of TMED2 using multiple public databases. These analyses included assessments of prognostic significance, gene mutations, pathway enrichment, single-cell sequencing analysis, immune characteristics, co-expressed gene PPI network analysis, as well as the therapeutic response of TMED2 in immunotherapy and small molecule sensitivity. Finally, we examined the role that TMED2 plays at the cellular level.

**Results:**

Our results show that the mRNA levels of TMED2 differ significantly between cancerous and normal tissues and are closely associated with cancer prognosis. Specifically, in CESC, MESO, LGG, and UVM, overexpression of TMED2 correlates with patient prognosis and various clinical pathological features. TMED2 is significantly associated with immune infiltration (including endothelial cells, neutrophils, dendritic cells, and eosinophils), immune checkpoints (CD274, HAVCR2, PDCD1LG2, and SIGLEC15), and signaling pathways (cell cycle and PI3K/Akt). Single-cell sequencing reveals that TMED2 is predominantly expressed in tumor cells of cervical cancer, glioma, and mesothelioma. Enrichment analysis shows that genes co-expressed with TMED2 are primarily involved in processes like endoplasmic reticulum stress and the ERAD pathway. Furthermore, cellular studies indicated that TMED2 expression promotes the growth, migration and invasion of glioma cells.

**Conclusion:**

Our integrated analysis suggests that targeting TMED2, along with its associated genes and signaling pathways, could represent a new strategy for cancer immune treatment.

## 1 Introduction

Cancer, as one of the key challenges in global public health, has been a central focus of medical research. With the advancing understanding of cancer pathogenesis, immunotherapy has emerged as a crucial strategy for cancer treatment (1–4). By activating the immune system to identify and destroy cancerous cells, immunotherapy offers new hope for cancer patients. However, the high heterogeneity of cancer results in significant variations in the efficacy of immunotherapy across different cancer types (5, 6). This emphasizes how vital it is to find new biomarkers to better predict patient responses to immunotherapy and optimize treatment strategies.

Transmembrane emp24 domain-containing protein 2 (TMED2) has gained increasing attention in recent years as a key molecule potentially involved in the process of tumorigenesis and development and progression (7, 8). TMED2 is the only member of the β-subfamily within the mammalian TMED family (9). All TMED family members share a similar domain architecture, including an N-terminal Golgi dynamics (GOLD) domain and coil-coiled (CC) domain, a transmembrane domain, and a C-terminal cytoplasmic domain (9). The intracellular physiological functions of TMED2 are complex, and one of its primary functions is mediating protein transportation from the endoplasmic reticulum (ER) to the Golgi apparatus (10). TMED2 binds to proteins destined for transport, facilitates their proper folding and packaging, and delivers them to the Golgi for further modification and processing (11, 12).

As part of the vesicle trafficking system, TMED2 regulates the formation, transport, and fusion of vesicles (13). By interacting with vesicle-associated proteins, it influences the dynamic properties of vesicles, ensuring efficient and accurate intracellular material transport. This regulation is vital for maintaining intracellular homeostasis and normal organelle functions (14). Previous research have revealed aberrant expression of TMED2 in specific cancer types (15). In ovarian cancer, high TMED2 expression has been connected with increased proliferation and invasion of cancer cells (16). Additionally, TMED2 expression has been linked to the development of cancerous cells in breast cancer (17) and head and neck squamous cell carcinoma (18). In the context of cancer, TMED2 may impact tumor growth, metastasis, and its interactions with the immune system. Studies have already analyzed its expression across various human cancer subtypes (8, 19). However, more comprehensive analyses are still lacking, such as protein interaction networks co-expressed with TMED2, single-cell sequencing analyses, and a detailed understanding of its mechanisms in tumor-immune interactions. Therefore, the potential roles and clinical applications of TMED2 in cancer remain to be explored further.

In this study, the prognostic and immune-related functions of TMED2 across pan-cancer were comprehensively examined by using public databases, including TCGA (The Cancer Genome Atlas), GTEX (Genotype-Tissue Expression Program), and CCLE (Cancer Cell Line Encyclopedia). We performed GSEA (Gene Set Enrichment Analysis) pathway enrichment analyses and explored the correlations between TMED2 levels and genetic mutation statuses in these cancers. Furthermore, TMED2’s co-expression in different cell types within the tumor microenvironment was validated through online datasets and single-cell sequencing analysis. We then performed Spearman correlation analysis to identify genes co-expressed with TMED2, followed by protein-protein interaction (PPI) network analysis and enrichment analyses using Gene Ontology (GO) and Kyoto Encyclopedia of Genes and Genomes (KEGG) pathways. We also predicted potential immunotherapy efficacy and drug sensitivity targeting TMED2 in these cancers. Finally, experimental validation was conducted to evaluate the impact of TMED2 knockdown on abnormal biological behaviors, such as proliferation, in glioma cells. In summary, TMED2 has promise as an effective target and a biomarker for predicting treatment responses in cancer therapy.

## 2 Materials and methods

### 2.1 Open data collection

TCGA RNA-seq data and metadata were obtained from the UCSC Xena platform (https://xenabrowser.net/). The scRNA-seq data including cervical cancer (GSE168652), glioma (GSE131928) and mesothelioma (GSE201925) was downloaded from GEO (https://www.ncbi.nlm.nih.gov/geo/). The drug-susceptibility and cell line DATA was downloaded from GDSC (https://www.cancerrxgene.org/), CCLE (https://sites.broadinstitute.org/ccle/) and CellMiner (https://discover.nci.nih.gov/cellminer/home.do).

### 2.2 Single-cell sequencing analysis

The R package (Seurat v5.2) were applied for scRNA-seq data integration and quality control (13). Cells were filtered based on the following criteria: nFeature_RNA > 200, percent.mt < 20, nCount_RNA > 800, and percent.hb < 5. Dimensionality reduction using Principal Component Analysis (PCA). The Harmony package was used for data integration. The FindClusters function was applied to cluster the cells together. Visualization of dimensionality reduction with UMAP functions. Marker genes utilized for the annotation of cell clusters were presented in **Supplementary Table S3**.

### 2.3 Research on the prognostic and immune roles of TMED2

The overall survival (OS) and progression free interval (PFI) were analyzed using the Kaplan-Meier (KM) curve. The IOBR package was used to assess the immunological landscapes of TMED2 (20). The reactions of TMED2 to immunotherapy and gene treatment in these tumors were examined using the TIDE (http://tide.dfci.harvard.edu) and ROC plotter (https://rocplot.org/) resources. The MuTarget (https://www.mutarget.com/analysis?type=target) was used to examine the association between TMED2 expression and various mutations in gene status within these malignancies. Using the clusterProfiler package, the Gene Set Enrichment Analysis (GSEA), Kyoto Encyclopedia of Genes and Genomes pathway analysis (KEGG) and Gene Ontology enrichment analysis (GO) was used to find rich signaling pathways (21). The scRNA-seq analysis was followed by the pipeline of Seurat (v5.2). The PPI-network was construct by the STRING (https://cn.string-db.org/) and the corresponding results were utilized by the STRINGdb package.

### 2.4 Cell culture

The cell lines HEK293T, U87 and U251 were obtained from the Stem Cell Bank, Chinese Academy of Sciences. And the cells were cultivated in DMEM (Gibco, #11965092) medium with 10% FBS (PAN Biotech, #ST30-3302).

### 2.5 Generation of cell lines

PEI (Sigma-Aldrich, #764604) was used as the transfection reagent, and pMD2.G (Addgene, #12259), psPAX2 (Addgene, #12260) and pLKO.1 shRNA vector were co-transfected into HEK293T cells. After transfection, cell supernatants were collected at 48 h and 72 h, respectively, and filtered using a 0.45 μm filter to remove cellular debris to obtain a viral suspension. Subsequently, the viral suspension was used to infect the target cells for 48 hours. To screen successfully infected cells, puromycin (2 μg/mL, MCE, #HY-K1057) was conducted to resistance screening for one to two weeks to obtain stable pLKO.1 shRNA expressing cell lines. TMED2 shRNA targeting sequence: GGACATCGACGTGGAGATTAC.

### 2.6 Western blotting

Cells were lysed in RIPA buffer (Epizyme Biotech, #PC101) with complete protease inhibitors (TargetMol, #C0001) and phosphatase inhibitors (Beyotime Biotechnology, #P1081 and P1086). Then cell debris was removed by centrifugation. Protein samples were separated by SDS-PAGE and transferred onto Immobilon-FL PVDF membrane (0.45 μm, Merck Millipore, #IPVH00005). After transfer, the membranes were blocked with 4% BSA for 1 h, followed by incubation with the indicated antibodies at 4°C overnight. The following day, the membranes were washed three times with TBST for 10 minutes each time and then incubated with the corresponding secondary antibody for 1 hour at room temperature. Finally, the target protein bands were developed using ECL chromogenic solution (Shandong Sparkjade Bio-technology Co., Ltd., #ED0015). Image J software was used to quantitatively evaluate the bands, using either β-actin or GAPDH to be the internal reference control. The antibodies used in the experiment are shown in **Supplementary Table S1**.

### 2.7 RT-qPCR

Total RNA was extracted using QIAGEN RNeasy Mini Kit (Qiagen, #74104) for RT-qPCR experiments. Subsequently, RNA was reverse transcribed into cDNA using HiScript III 1st Strand cDNA Synthesis Kit (Vazyme Biotech Co., Ltd., #R312). Mixing cDNA template obtained from reverse transcription with specific primers and SYBR Green qPCR mix (Shandong Sparkjade Bio-technology Co., Ltd., #AH0104) to construct the reaction system. The reaction system was constructed. The reaction was performed on an ABI-7500 Real-time PCR system. The relative expression levels of the target genes were evaluated by the 2-ΔΔCt method, and β-actin was used as the internal reference gene for normalization. The primers were synthesized by RuiboBio (Qingdao, China) displayed in **Supplementary Table S2**.

### 2.8 Tumor phenotype analysis

Cell viability assay: Cells were treated and inoculated in 96-well plates, each well was filled with CCK-8 solution (Yeasen, #40203ES76), which was then incubated for 1.5 hours at 37°C. Cell viability was evaluated by measuring absorbance at 450 nm.

Colony formation assay: 500 cells per well were inoculated in six-well plates for about 10–15 days. The colonies underwent fixation using 4% paraformaldehyde (PFA), Subsequently, the colonies were subjected to staining with 0.1% crystal violet and photographed.

Cell migration assay: 3 × 10^4^ cells were digested with trypsin, suspended in serum-free medium, and added to the upper chamber of Transwell (pore size 8 μm, Corning). The lower chamber was filled with a medium that included serum. After removal of nonmigrating cells, they were fixed with 4% PFA, colored with 0.1% crystalline violet, and photographed for evaluation.

### 2.9 Statistical analysis

Data are shown as the mean ± standard deviation (M ± SD). Statistical analyses were performed using GraphPad Prism 9, with significance levels defined as *P < 0.05, **P < 0.01 and ***P < 0.001. R version 4.3.1 was used to make all bioinformatic and statistical analyses. The Wilcoxon rank-sum test was accustomed to estimate levels of TMED2 expression based on pathological features. Log-rank tests were used to calculate the survival probability using Kaplan-Meier survival curves. Student’s t-test was performed using parametric tests (unpaired two-tailed Student’s t-test) and nonparametric testing (Mann-Whitney test), determined by whether test assumptions were met. The figure legends of every dataset list the precise tests that were employed.

## 3 Results

### 3.1 TMED2 expression and prognosis

**Figure 1:**
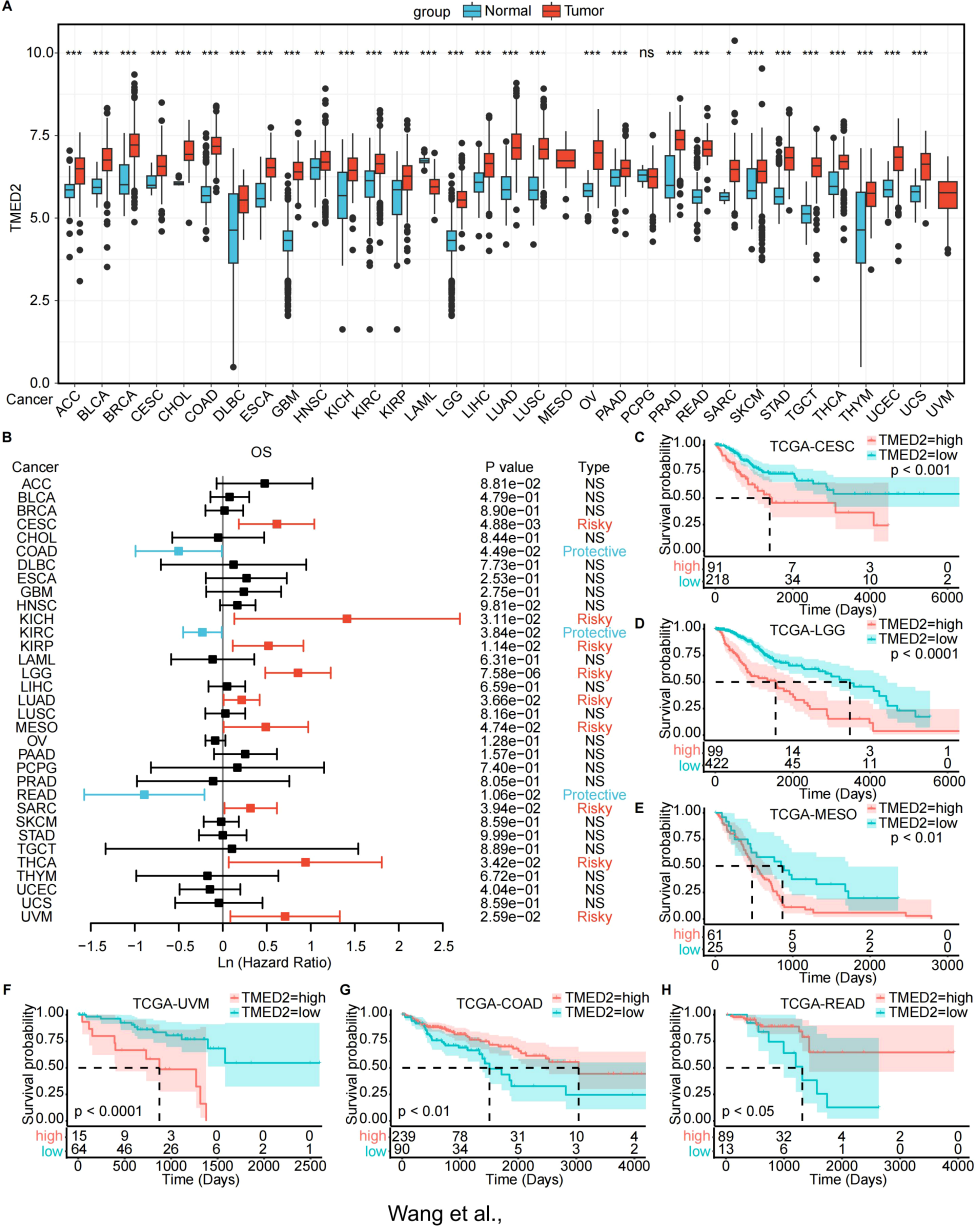
Expression landscape and prognostic analysis of TMED2. **(A)** TMED2 expression in the normal and tumor samples analyzed by GTEx and TCGA dataset. **(B)** Forest plot of survival analysis of TMED2 in OS. **(C-H)** The Survival analysis of TMED2 on OS by the KM analysis: CESE **(C)**, LGG. **(D)**, MESO **(E)**, UVM **(F)**, COAD **(G)**, READ **(H)**. *P < 0.05, **P < 0.01, ***P < 0.001, ns, not significant.

First, we examined the mRNA expression levels of paired normal and malignant tissues using TCGA database. The findings demonstrated that TMED2 expression was increased in BLCA, BRCA, COAD, ESCA, HNSC, KIRC, KIRP, LIHC, LUAD, LUSC, PRAD, and STAD compared to their normal tissue counterparts (**Supplementary Figure S1A**). Subsequently, we combined the TCGA and GTEx databases for a more comprehensive analysis of TMED2 mRNA expression in pan-cancer. TMED2 was significantly upregulated in a wide range of cancers, including ACC, BLCA, BRCA, CESC, CHOL, COAD, DLBC, ESCA, GBM, HNSC, KICH, KIRC, KIRP, LGG, LIHC, LUAD, LUSC, OV, PAAD, PRAD, READ, SARC, SKCM, STAD, TGCT, THYM, UCEC, and UCS, while only LAML exhibited downregulated TMED2 expression (**Figure 1A**). Additionally, we analyzed the expression of TMED2 across different cancer stages and tumor histology. The results showed that in the clinical staging of CESC and pathological tumor staging of MESO, the expression of TMED2 was higher in stages I and IV compared to stages II and III (**Supplementary Figures S1B, C**). In the histological grading of LGG, TMED2 expression was higher in grade G3 than in grade G2 (**Supplementary Figure S1D**). Among histological classifications, TMED2 expression was highest in the adenosquamous carcinoma of CESC and the epithelioid subtype of UVM (**Supplementary Figures S1E, F**). These results showed that TMED2 might be involved in the development and progression of different cancers.

Next, we investigated the prognostic significance of TMED2 across different cancer types. TMED2 was found to act as a risk factor in the progression of many tumors. Univariate Cox regression analysis revealed that increased TMED2 expression was strongly related with poor overall survival (OS) in CESC, KICH, KIRP, LGG, LUAD, MESO, SARC, THCA, and UVM (**Figure 1B**). Additionally, elevated TMED2 expression predicted shorter progression-free intervals (PFI) in ACC, CESC, LGG, MESO, and UVM (**Supplementary Figure S3A**). Furthermore, In CESC, KICH, KIRP, LGG, MESO, and UVM, increased TMED2 expression was associated with decreased disease-specific survival (DSS) (**Supplementary Figure S3H**). We also conducted OS analysis based on the optimal cutoff for TMED2 expression (**Figures 1C-H**; **Supplementary Figures S2A-F**) and PFI analysis (**Supplementary Figures S3B-G**). These analyses collectively indicate that high TMED2 expression is a significant adverse prognostic factor for patient survival, particularly in cancers such as CESC, LGG, MESO, and UVM.

### 3.2 Mutation analysis of TMED2 in pan-cancer

**Figure 2:**
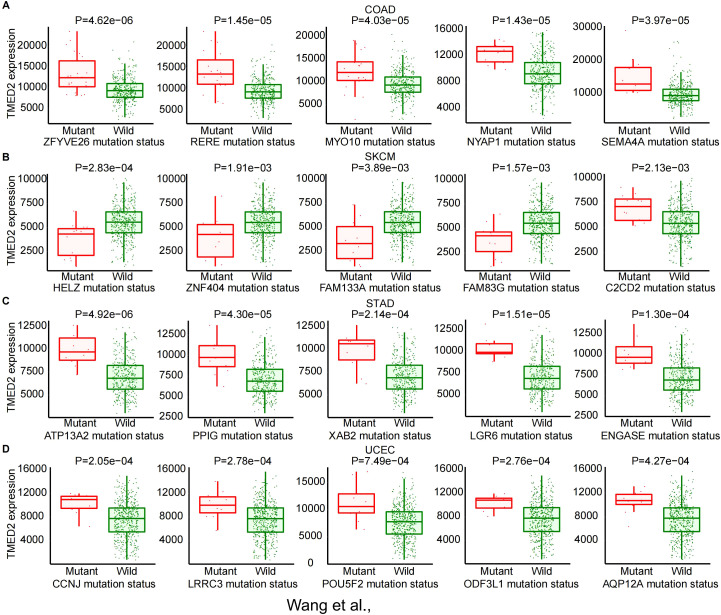
The correlation between TMED2 expression and gene mutation status: COAD **(A)**, SKCM **(B)**, STAD **(C)**, UCEC **(D)**.

Genetic mutations are a fundamental cause of tumorigenesis. Using the MuTarget database, we examined the association between TMED2 expression and genetic mutations in a rage of cancer types. The findings revealed that in COAD, higher TMED2 expression was observed in the mutant groups of ZFYVE26, RERE, MYO10, NYAP1, and SEMA4A compared to their wild-type counterparts (**Figure 2A**). In SKCM, TMED2 expression was higher in the wild-type groups of HELZ, ZNF404, FAM133A, and FAM83G compared to their mutant groups, while the opposite trend was observed for C2CD2 (**Figure 2B**). In STAD, the wild-type groups of multiple genes, including ATP13A2, PPIG, XAB2, LGR6, and ENGASE, exhibited lower TMED2 expression compared to their mutant counterparts (**Figure 2C**). In UCEC, the mutant groups of CCNJ, LRRC3, POU5F2, ODF3L1, and AQP12A showed higher TMED2 expression than the wild-type groups (**Figure 2D**). Additionally, higher TMED2 expression was observed in the mutant groups of TYK2 in BLCA, ARMC9 in CESC, TGM4 and KCNK18 in LUAD, SPON1 and CEP128 in LUSC, and TRDN in SARC compared to their respective wild-type groups. Conversely, the wild-type groups of NBPF10 in CESC, CHD4 in KIRC, SMARCA4 and NFE2L2 in KIRP, OBSCN in SARC, and EPHA7 in OV exhibited higher TMED2 expression than their mutant counterparts (**Supplementary Figure S4**). These mutated genes are involved in critical cellular processes, especially in tumor cells. For instance, SMARCA4 regulates gene expression by remodeling chromatin structure (22), NFE2L2 acts as a key redox transcription factor within cells (23, 24), and LGR6 is a G-protein-coupled receptor 6 that contains leucine-rich repeats (25). These findings highlight the close relationship between TMED2 and genetic mutations in pan-cancer.

### 3.3 Immunological characteristics of TMED2 in the tumor microenvironment

**Figure 3:**
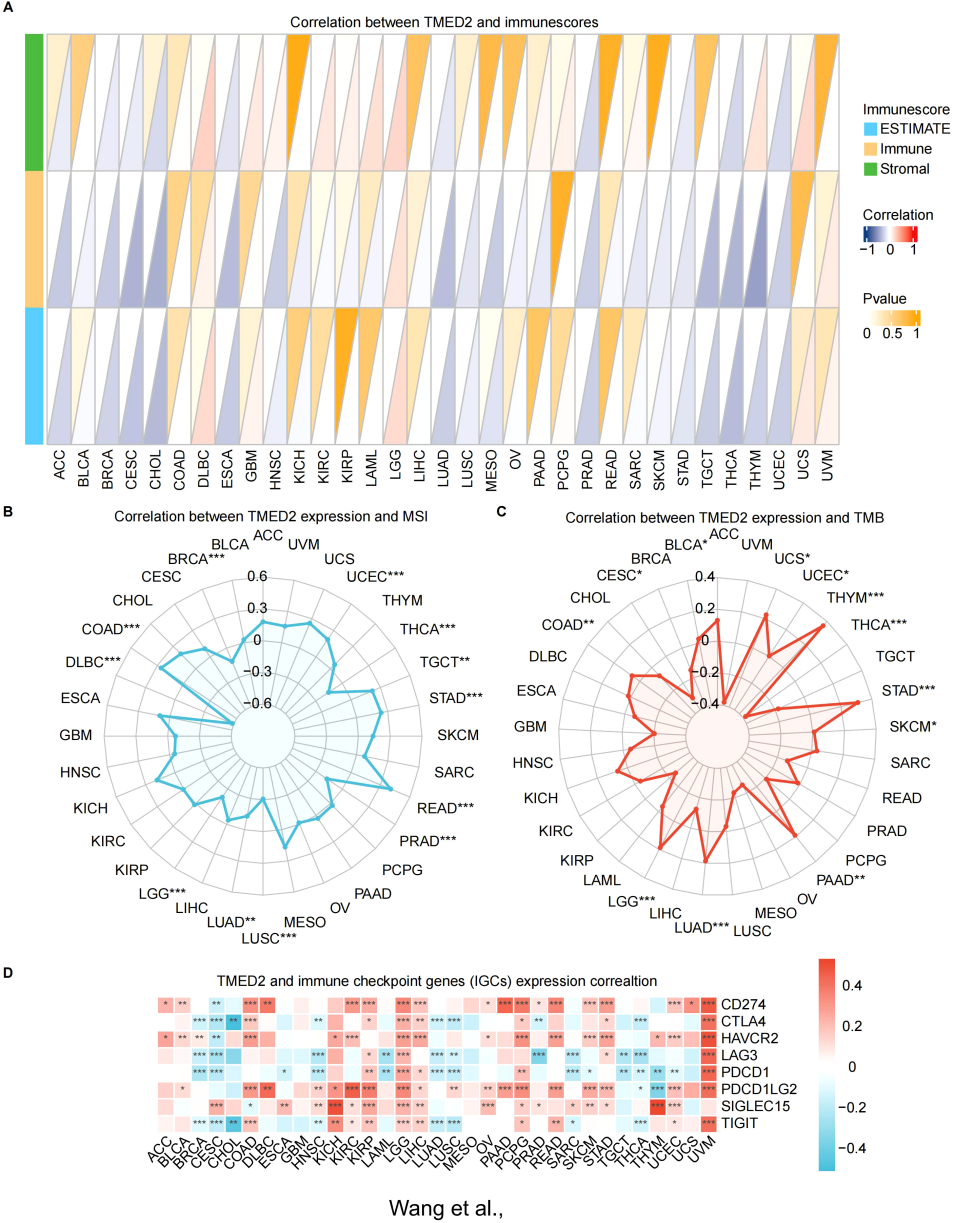
Relationship of TMED2 expression level with tumor immune characteristics. **(A)** The relationship between TMED2 expression and three scores (immune, estimate, and stromal) in TCGA cancers using ESTIMATE analysis. **(B)** Correlation between TMED2 expression and TMB displayed by the radar chart. **(C)** Correlation between TMED2 expression and MSI displayed by the radar chart. **(D)** Relationship between TMED2 expression and various immune checkpoints. *P < 0.05, **P < 0.01, ***P < 0.001, ns, not significant.

By stimulating the patient’s immune system to identify and combat cancer cells, immunotherapy has become a major focus in cancer treatment research (26–28). We examined several immunological characteristics of TMED2 within the tumor immune microenvironment (TIME) in order to investigate the connection between TMED2 and immunotherapy. Correlations between TMED2 expression levels and stromal, immunological, and ESTIMATE scores were assessed using the ESTIMATE algorithm for a variety of cancer types. DLBC, KIRC, LAML, LGG, THYM, and UCS were the leading six malignancies with a positive connection between TMED2 levels and stromal scores. Among malignancies, the strongest positive correlation between TMED2 levels and immune scores was observed in LGG. The top four cancers with a positive correlation between TMED2 levels and ESTIMATE scores were DLBC, LGG, UCS and UVM (**Figure 3A**).

Subsequently, we applied four immune infiltration algorithms, including CIBERSORT, MCPCOUNTER, QUANTISEQ and TIMER, to examine the relationship between TMED2 and immune or stromal cells in various cancers (**Supplementary Figure S5**). The findings showed a positive relationship between TMED2 expression and the infiltration levels of endothelial cells, neutrophils, dendritic cells, and eosinophils in most cancers. In contrast, the expression of TMED2 was negatively correlated with the infiltration levels of monocyte.

We also investigated the relationship between TMED2 and dynamic immune-related features, including two cutting-edge immunotherapy biomarkers: microsatellite instability (MSI) and tumor mutation burden (TMB). TMED2 had a positive correlation with MSI in UCEC, TGCT, STAD, READ, and COAD (**Figure 3B**). Additionally, TMED2 had a positive correlation with TMB in UCS, THYM, STAD, PAAD, LUAD, LGG, COAD, and BLCA, but a negative correlation with TMB in THCA and CESC (**Figure 3C**).

Given the notable correlations between TMED2 and immune cells, we examined its relationship with different classical immune checkpoints of cancers. The findings showed a substantial positive correlation between TMED2 levels and immune checkpoint molecules, including SIGLEC15, PDCD1LG2, HAVCR2, and CD274, in KIRC, LGG, LIHC, OV, PCPG, SKCM, STAD, UCEC and UVM (**Figure 3D**).

These results imply that TMED2 might play a pivotal role in the tumor immune microenvironment by regulating the characteristics of immune cells or the expression levels of immunoregulatory genes, thereby influencing cancer growth and response to treatment.

### 3.4 Functional enrichment analysis of TMED2

**Figure 4:**
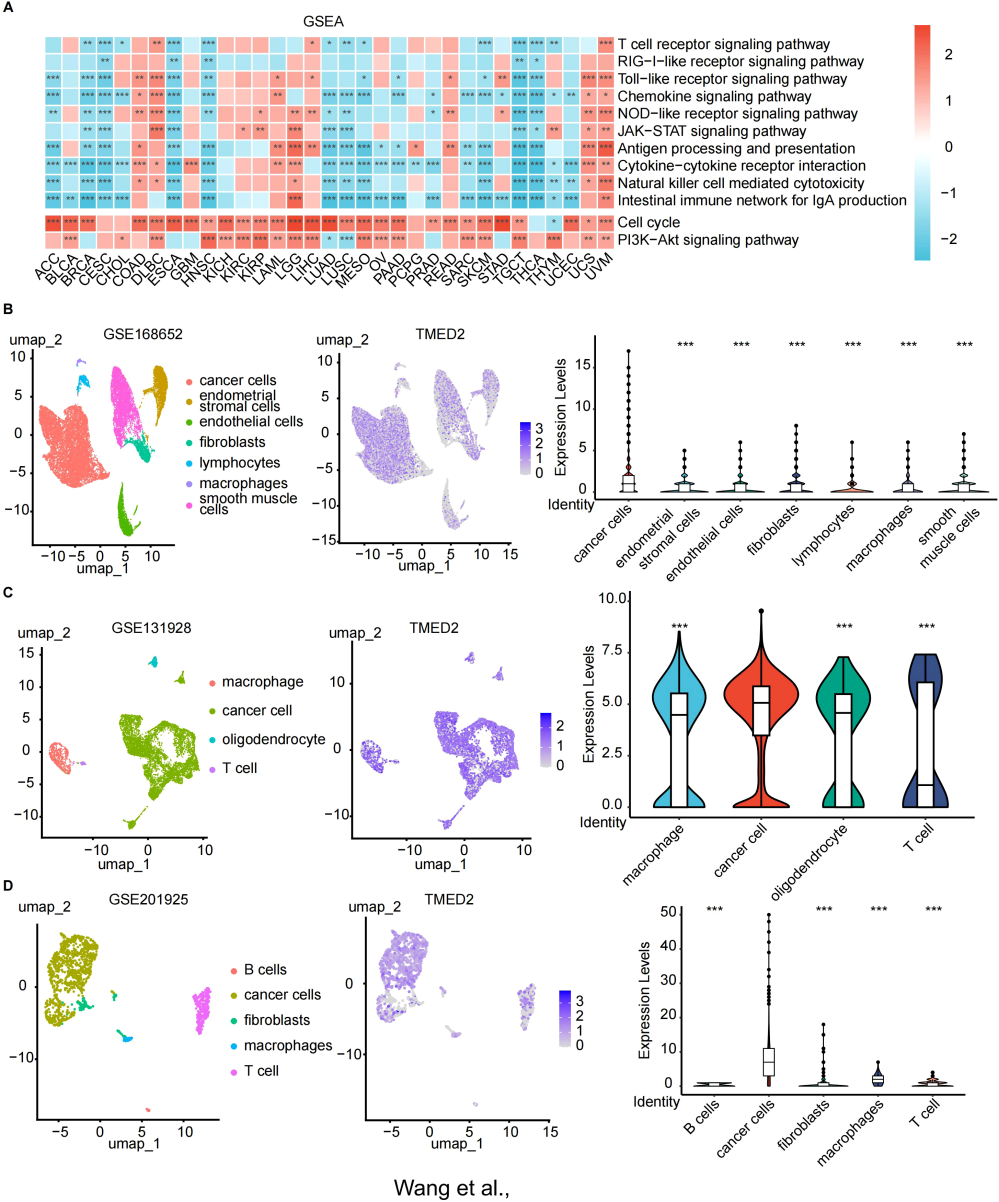
The relationship of TMED2 expression level with Gene Set Enrichment Analysis (GSEA) and single-cell sequencing. **(A)** Heatmap of the TMED2 pathway enrichment study. **(B-D)** Single-cell sequencing analysis of TMED2 expression in tumor tissues of cervical cancer **(B)**, glioma **(C)**, and MESO **(D)**. *p < 0.05, **p < 0.01, ***p < 0.001.

To further investigate the potential mechanisms underlying TMED2 function, we performed GSEA analysis in various cancers. In the high TMED2 expression groups of multiple tumor types, pathways related to cell growth and immunity were significantly enriched. Across nearly all cancers, the activation of the PI3K/Akt and cell cycle signaling pathways was positively connected with TMED2 expression (**Figure 4A**). The PI3K/Akt signaling pathway is essential for a number of physiological processes, such as cell growth, proliferation, differentiation, and migration. Additionally, TMED2 was significantly associated with NOD-like receptor signaling and antigen processing and presentation pathways in COAD, LGG, LIHC, READ, UCS, and UVM. These findings suggest that TMED2 participates in a number of biological processes and could be crucial to the treatment of cancer.

### 3.5 Relationship between TMED2 and tumor cells via single-cell sequencing

Next, we examined TMED2 expression in tumor, immune, and stromal cells from a variety of solid tumors types, including cervical cancer, glioma, and mesothelioma (**Figures 4B-D**). TMED2 demonstrated significant co-expression across tumor cells. Notably, among these cancer types, TMED2 expression was highest in tumor cells, underscoring its potential role in tumor biology.

### 3.6 Predicting the immunotherapeutic value of TMED2

**Figure 5:**
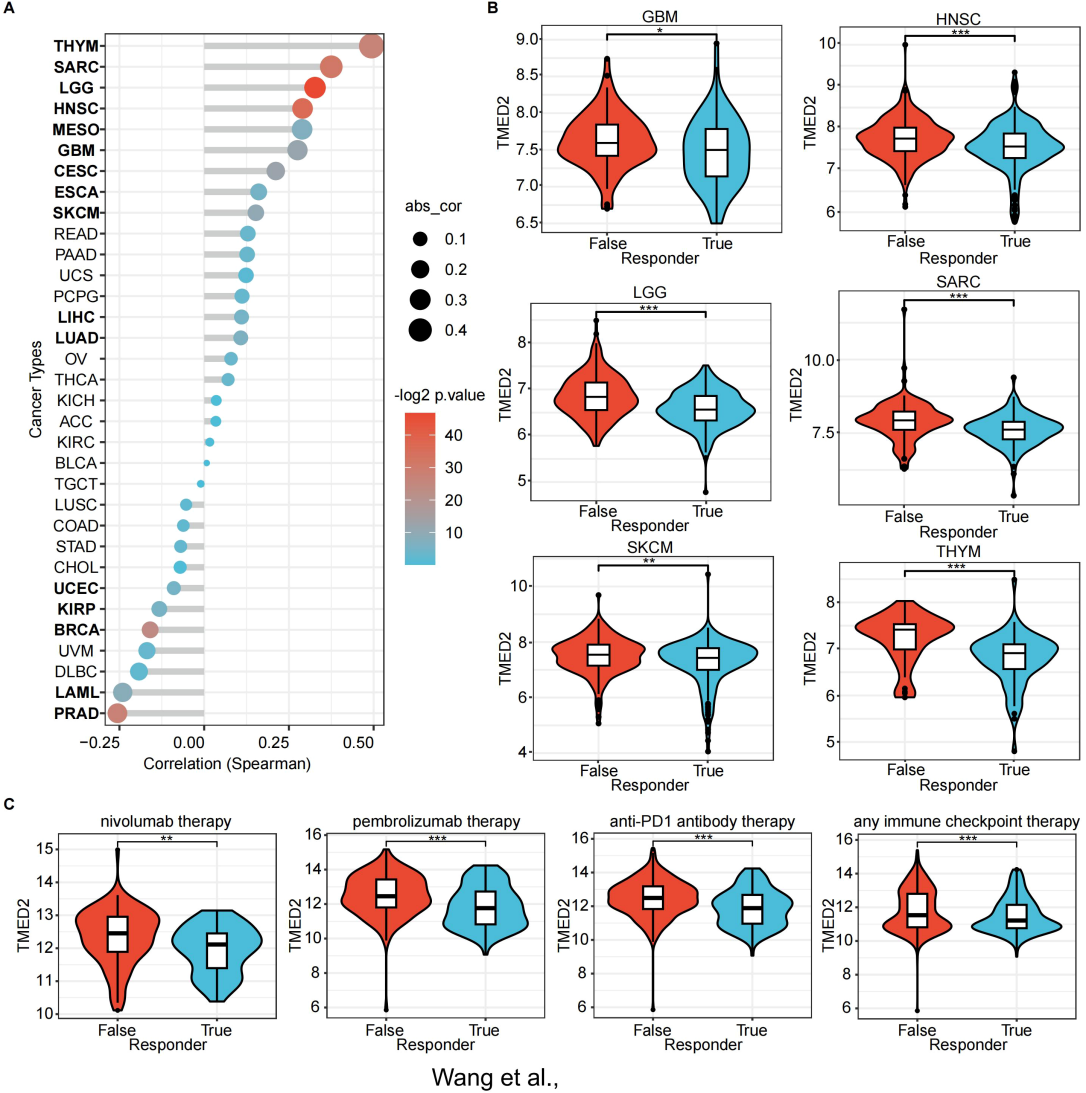
The association of TMED2 expression with immunotherapy response and Tumor Immune Dysfunction and Exclusion (TIDE) scores. **(A)** The association between TMED2 expression and TIDE score. **(B)** The distribution of TIDE scores across TMED2 high and low expression groups in various tumors. **(C)** The expression of TMED2 in response and non-response groups of different immunotherapeutic cohorts. *P < 0.05, **P < 0.01, ***P < 0.001, ns, not significant.

To systematically explore the potential of TMED2 as an immunotherapy target, we predicted immunotherapeutic responses based on public databases. TIDE scores, a usual and trustworthy biomarker for predicting immune therapy response, were calculated for patients with different TMED2 expression levels. TMED2 expression and TIDE scores were positively correlated in the majority of solid malignancies, especially in THYM, SARC, LGG, and HNSC (**Figure 5A**). In some solid tumors, non-responders to immunotherapy exhibited higher TMED2 expression levels, suggesting reduced therapeutic benefits from immunotherapy (**Figure 5B**).

Using the ROC Plotter dataset, we also analyzed TMED2 expression’s predictive value for therapeutic responses in NSCLC, SKCM, HNSC, GBM, and BLCA. The results indicated that higher TMED2 expression was observed in non-responders to immunotherapies, including nivolumab, pembrolizumab, anti-PD-1, and other immune checkpoint inhibitors (**Figure 5C**). These findings highlight TMED2 as a potential biomarker for predicting immunotherapy outcomes.

### 3.7 PPI network and enrichment pathways of TMED2 co-expressed genes

**Figure 6:**
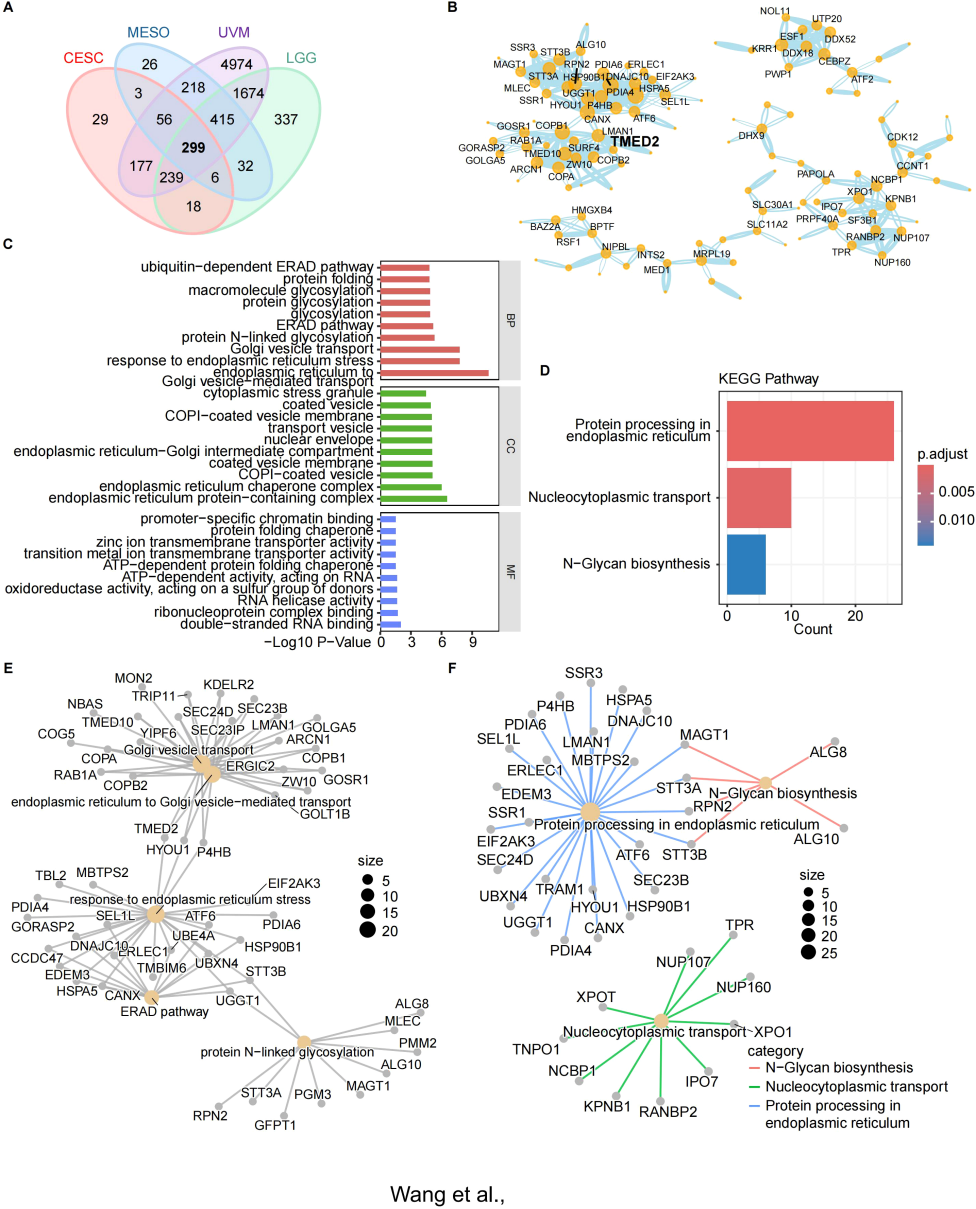
The relationship between the expression of TMED2 and the expression of other genes. **(A)** Venn diagram of genes co-expressed with TMED2. **(B)** Core nodes of the PPI network of TMED2 and co-expressed genes. **(C-F)** Bar chart **(C)** and network diagram **(E)** for the Gene Ontology (GO) enrichment analysis of TMED2 and other genes; Bar chart **(D)** and network diagram **(F)** for the Kyoto Encyclopedia of Genes and Genomes (KEGG) enrichment analysis of TMED2 and other genes.

To explore whether TMED2 functions independently in cancer or collaborates with other genes for co-regulation, we attempted to construct its potential regulatory network. For this, we selected four cancer types that exhibited poor prognosis in the previous analysis, including CESC, MESO, LGG, and UVM, for in-depth investigation. Using Spearman correlation analysis, we identified genes that were co-expressed with TMED2, setting the criteria as a correlation coefficient (R) > 0.5 and P < 0.05. We then performed a Venn diagram analysis to identify the intersection of these genes, ultimately selecting 299 genes co-expressed with TMED2 (**Figure 6A**). Subsequently, we utilized the STRING database to perform PPI network analysis on these 299 genes and TMED2 (**Supplementary Figure S6**), retaining the core nodes (**Figure 6B**). We then conducted GO and KEGG enrichment analyses for these genes (**Figures 6D, F**).

The results of the GO enrichment analysis revealed that multiple biological processes, cellular components, and molecular functions were significantly correlated with TMED2 expression. Notably, processes such as endoplasmic reticulum-associated degradation (ERAD), glycosylation modification, and endoplasmic reticulum stress were identified as key pathways (**Figure 6C**). In the enrichment network, TMED2 was found to be significantly associated with biological processes such as Golgi vesicle transport, transport from the endoplasmic reticulum to the Golgi apparatus, ER stress, the ERAD pathway, and N-linked glycosylation (**Figure 6E**). KEGG Enrichment Analysis: The KEGG enrichment results showed a significant positive correlation between TMED2 expression and protein processing in the endoplasmic reticulum as well as nucleocytoplasmic transport (**Figures 6D, F**).

Notably, Golgi transport protein 1B (GOLT1B) was upregulated in most cancer tissues and was associated with immune cell infiltration, particularly the infiltration of T helper type 2 (Th2) cells (29). Additionally, the activation of transcription factor 6 (ATF6) following ER stress inhibits the expression of ΔNp63α through the GRP78-AKT1-FOXO3a signaling pathway, thereby promoting breast cancer metastasis (30). Oligosaccharyltransferase subunit (STT3B) was shown to stabilize Epiregulin via N-glycosylation, which is crucial for PD-L1 upregulation and immune escape in head and neck squamous cell carcinoma (31).

These findings suggest that TMED2 does not act independently in cancer, but rather works synergistically with multiple genes within the same subcellular structures. It participates in various critical biological processes and signaling pathways, thereby influencing cancer development, progression, and immune evasion.

### 3.8 Drug sensitivity prediction of TMED2

**Figure 7:**
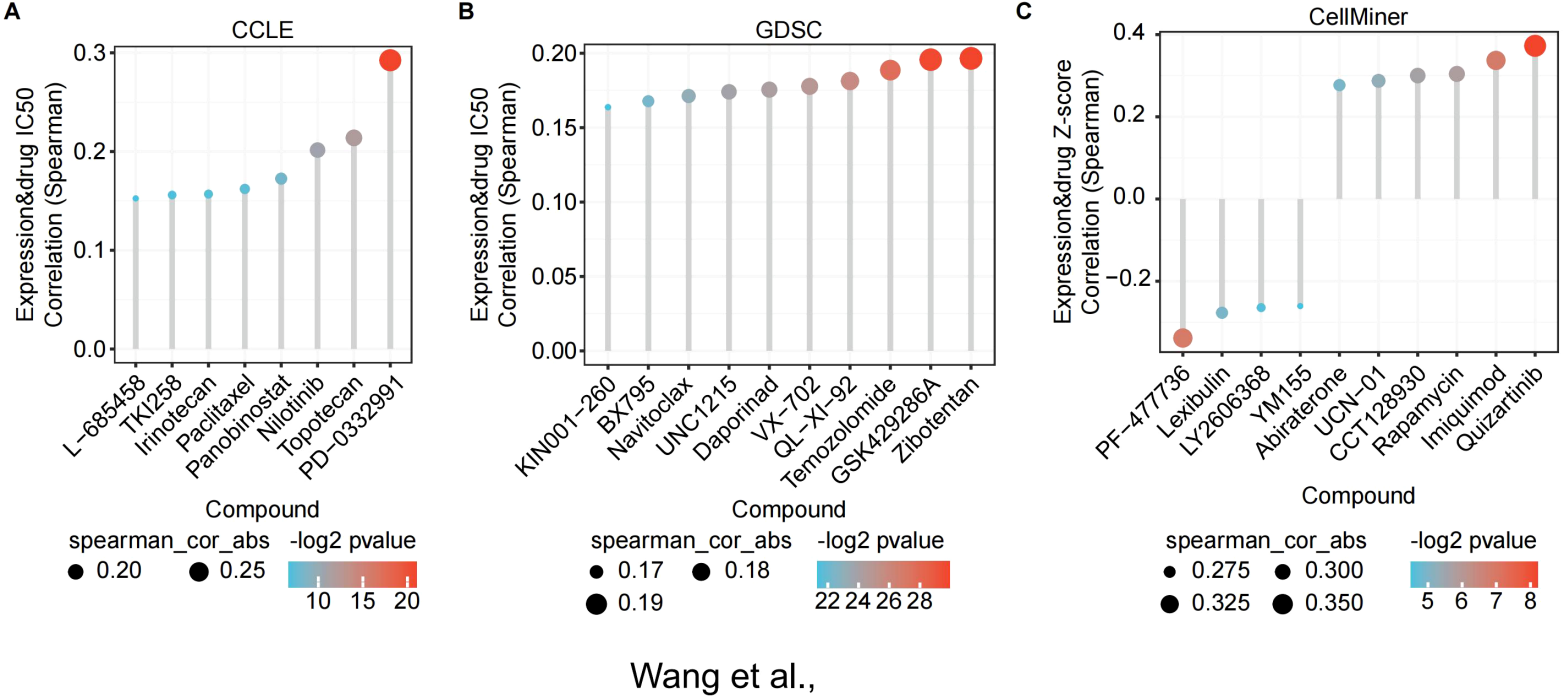
The correlation between TMED2 expression and the small molecule drugs. The correlation between TMED2 expression and the small molecule drugs through CCLE **(A)**, GDSC **(B)**, and CellMiner **(C)** datasets.

The above analysis indicates that TMED2 exhibits significant abnormal expression in various cancers and is closely associated with key biological processes such as tumor proliferation, differentiation, and immune evasion. These findings suggest that TMED2 could be a potential therapeutic target. Building on this, we further explored the correlation between small molecule drugs and TMED2 expression, aiming to provide more specific clues and directions for the development of drugs targeting TMED2. According to the CCLE dataset, Topotecan, Nilotinib, and PD-0332991 were the most three resistant drugs (**Figure 7A**). The top three resistant drugs associated with high TMED2 expression were Zibotentan, GSK429286A, and Temozolomide, according to the GDSC (Genomics of Drug Sensitivity in Cancer) dataset (**Figure 7B**). From the CellMiner dataset, the top four sensitive compounds were identified as Quizartinib, Imiquimod, Rapamycin, and CCT128930 (**Figure 7C**). These small molecules play antitumor roles in various cancers. Paclitaxel promotes tubulin polymerization into stable microtubules, inhibiting depolymerization and inducing mitotic arrest and apoptosis, making it a first-line treatment for breast cancer (32). Quizartinib, an FMS - like tyrosine kinase 3 (FLT3) inhibitor, specifically blocks FLT3 receptor tyrosine kinase activity, suppressing downstream signaling pathways and preventing FLT3-ITD-mutant acute myeloid leukemia cells from proliferating and surviving while inducing apoptosis (33). Our findings show that TMED2 expression is closely connected with drug treatment efficacy. The results of drug sensitivity analysis suggest that TMED2 may regulate these related pathways.

### 3.9 TMED2 promotes glioma cell malignancy

According to our findings, TMED2 has high expression in malignancies. Results from single-cell sequencing further demonstrated that tumor cells might express more TMED2. Additionally, TMED2 expression has positive correlation with immunoregulatory pathways, like the cell cycle and PI3K/Akt signaling pathways, which influence tumor growth and expansion. These findings suggest that TMED2 might function as an oncogene in tumorigenesis and progression. We used U87 and U251 glioma cells with strong TMED2 expression in our *in vitro* experiments to confirm this hypothesis.

**Figure 8:**
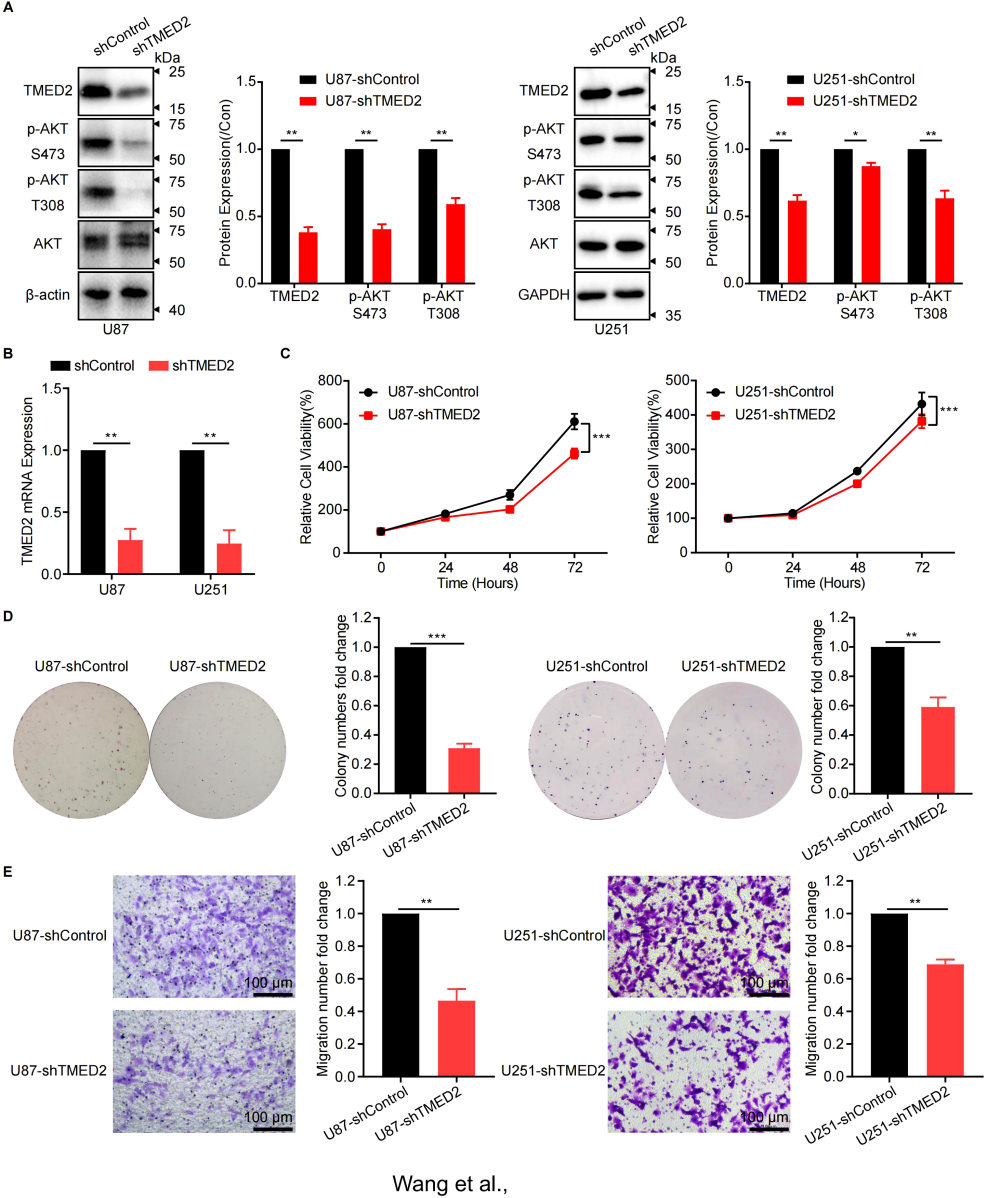
Knockdown of TMED2 inhibited the proliferation and migration ability and AKT signaling pathway of glioma cells. **(A)** The expression of TMED2 and AKT signal in TMED2 knock-down cells was detected by WB in U251 and U87 cells (Data represent three independent experiments, using unpaired two-tailed Student’s t-test). **(B)** The expression of TMED2 in TMED2 knock-down cells was detected by RT-qPCR in U251 and U87 cells. **(C)** The proliferation of U251 and U87 cells were evaluated by CCK8 assay (Data represent six independent experiments, using one-way ANOVA followed by Tukey’s multiple comparisons tests). **(D)** The colony formation of U251 and U87 cells (Data represent three independent experiments, using unpaired two-tailed Student’s t-test). **(E)** Cell migration of U251 and U87 cells was detected by the transwell assay (Data represent three independent experiments, using unpaired two-tailed Student’s t-test). Scale bars = 100 μm. *P < 0.05, **P < 0.01, ***P < 0.001, ns, not significant.

First, we knocked down TMED2 expression in U87 and U251 cells. The knockdown efficiency was assessed utilizing RT-qPCR and western blot (WB) analysis, which showed a substantial reduction in the levels of TMED2 mRNA and protein (**Figures 8A, B**). Phosphorylation of AKT was also downregulated, while total AKT protein levels remained unchanged compared to control cells (**Figure 8A**). This finding indicates that TMED2 probably enhances the AKT signaling pathway, consistent with the GSEA enrichment results mentioned earlier.

Next, tumor phenotypes were assessed. CCK-8 assays demonstrated that TMED2 knockdown significantly suppressed glioma cell proliferation (**Figure 8C**), a finding corroborated by reduced colony size in the plate colony formation assay (**Figure 8D**). These results suggest that TMED2 promotes glioma cell growth. Cell migration and invasion, critical factors in cancer metastasis and recurrence, were evaluated using Transwell assays (**Figure 8E**). TMED2 knockdown greatly decreased the number of tumor cells migrating to the lower chamber, indicating that TMED2 enhances the migration and invasion capabilities of glioma cells.

## 4 Discussion

Research to date has showed that TMED2 plays a crucial role in cell division and proliferation, particularly in cancerous tumors (16–18). However, previous research has primarily concentrated on TMED2’s function in specific cancer types, leaving its molecular characteristics in pan-cancer contexts underexplored. This study provides a comprehensive and in-depth investigation into the association between TMED2 expression and immunotherapy response in various cancers for the first time.

In our study, we employed a multidimensional approach leveraging extensive datasets. On the one hand, we examined TMED2 mRNA expression levels in cancer cell lines and tumor samples. Using TCGA and GTEX datasets, we observed a striking phenomenon: In comparison to normal samples, TMED2 expression was substantially elevated in almost all cancer cell lines. Single-cell sequencing further confirmed the high expression of TMED2 in tumor cells from cervical cancer, glioma, and mesothelioma. And its expression levels also varied across different cancer stages and tumor grade classifications. Additionally, analysis of the MuTarget dataset revealed disparities in the expression of TMED2 between mutant and wild-type (WT) groups in various cancers, shedding light on potential mutation mechanisms affecting TMED2 expression during tumorigenesis.

On the other hand, after clarifying the expression characteristics of TMED2 in cancer, we focused on exploring its potential as a therapeutic target for immune therapy in various solid cancers within the TIME. The results revealed clinically significant trends: patients with higher TMED2 expression levels derived less benefit from immunotherapies, including anti-PD-1/PD-L1 agents, nivolumab, pembrolizumab, and immune checkpoint inhibitors. To further explore the link between TMED2 and immunotherapy efficacy, we systematically evaluated the relationships between TMED2 and classical immune therapy biomarkers like MSI, TMB and TIDE scores. TMED2 was positively correlated with TMB in UCS, THYM, STAD, PAAD, LUAD, LGG, COAD, and BLCA but negatively correlated in THCA and CESC. TIDE scores, a validated predictive marker of immunotherapy response with high accuracy (34), were higher in patients with elevated TMED2 expression in most cancers, strongly suggesting that these patients may experience reduced immunotherapy efficacy.

The main goal of immunotherapy is to block immunological checkpoints. PD-1/PD-L1 and CTLA-4/B7 are two classical immune checkpoint pathways that that adversely affect T-cell immunological activity, especially during critical periods of T-cell activation and proliferation (35–37). Our study examined the correlations between TMED2 and different immune checkpoints across tumors. TMED2 expression was positively connected with important immune checkpoint markers such as CD274, HAVCR2, PDCD1LG2, and SIGLEC15 in numerous cancer types. However, we also observed that in certain cancers, such as CESC, LUAD, and THCA, many immune checkpoint molecules exhibited a negative correlation with TMED2 expression. This may suggest the complexity of different tissues, indicating that the expression of TMED2 is associated with the infiltration or activation of immune cells in various tissues.

In this study, we made two important discoveries. First, we utilized KEGG (Kyoto Encyclopedia of Genes and Genomes) analysis to investigate the role of TMED2 in tumors and explored its potential molecular mechanisms in cancer. We identified pathways like the cell cycle and PI3K/Akt signaling as being significantly associated with TMED2-mediated tumor immunity. This may be related to TMED2’s role in protein transport (38–40). Furthermore, our previous studies reported that TMED2 enhances EGFR-AKT signaling in glioma by participating in EGFR recycling (41). These findings suggest that targeting TMED2 could suppress tumorigenesis by modulating these pathways.

Second, by constructing the potential regulatory network of TMED2, we revealed its synergistic action in various cancers. The results showed that TMED2, in collaboration with multiple genes, is involved in key biological processes such as endoplasmic reticulum-associated protein degradation, glycosylation modifications of proteins and other macromolecules, and endoplasmic reticulum stress. It is also significantly associated with signaling pathways like protein processing and nucleocytoplasmic transport. These findings suggest that TMED2 does not function independently in cancer but influences cancer initiation, progression, and immune evasion through its collaborative regulation with other genes. This discovery provides new insights into understanding the biological functions of TMED2 in cancer.

The above findings highlight the critical role of TMED2 as a potential therapeutic target, providing a theoretical foundation for drug development based on TMED2. With the rapid advancement of bioinformatics technologies, identifying optimal personalized treatment medications from common databases and computational modes has become a burgeoning trend in oncology research (42, 43). In this study, we discovered a range of small-molecule compounds that are linked to the expression of TMED2, which notably includes resistant compounds (Zibotentan, GSK429286A, Temozolomide, PD-0332991, Topotecan, Nilotinib) and sensitive compounds (Quizartinib, Imiquimod, Rapamycin, CCT128930). These findings provide potential directions for future TMED2-targeted drug development.

Our study deeply investigated TMED2’s function in tumor immunology from a pan-cancer standpoint and verified that glioma cell proliferation and invasion are inhibited by TMED2 expression suppression. To confirm TMED2’s function in cancer and clarify its mechanisms as a target for diagnosis and treatment, more *in vitro* and *in vivo* research is necessary.

## 5 Conclusion

In this study, we performed a preliminary yet systematic analysis of TMED2 with gene mutations, pathway enrichment, classical immunotherapy biomarkers, drug treatment in pan-cancer. Our results demonstrate TMED2’s enormous promise as a therapeutic target, its crucial role in immunity to tumors, and its potential as a prognostic biomarker for several cancer types.

## Data Availability

The datasets presented in this study can be found in online repositories. The names of the repository/repositories and accession number(s) can be found below: TCGA RNA-seq data and metadata were obtained from the UCSC Xena platform (https://xenabrowser.net/). The scRNA-seq data including cervical cancer (GSE168652), glioma (GSE131928) and mesothelioma (GSE201925) was downloaded from GEO (https://www.ncbi.nlm.nih.gov/geo/). The drug-susceptibility and cell line DATA was downloaded from GDSC (https://www.cancerrxgene.org/), CCLE (https://sites.broadinstitute.org/ccle/) and CellMiner (https://discover.nci.nih.gov/cellminer/home.do).

## Glossary

TCGA: The Cancer Genome Atlas
CCLE: Cancer Cell Line Encyclopedia
GTEx: Genotype-Tissue Expression Program
ACC: Adrenocortical carcinoma
BLCA: Bladder Urothelial Carcinoma
BRCA: Breast invasive carcinoma
CESC: Cervical squamous cell carcinoma and endocervical adenocarcinoma
CHOL: Cholangiocarcinoma
COAD: Colon adenocarcinoma
DLBC: Lymphoid Neoplasm Diffuse Large B-cell Lymphoma
ESCA: Esophageal carcinoma
GBM: Glioblastoma multiforme
HNSC: Head and Neck squamous cell carcinoma
KICH: Kidney Chromophobe
KIRC: Kidney renal clear cell carcinoma
KIRP: Kidney renal papillary cell carcinoma
LAML: Acute Myeloid Leukemia
LGG: Brain Lower Grade Glioma
LIHC: Liver hepatocellular carcinoma
LUAD: Lung adenocarcinoma
LUSC: Lung squamous cell carcinoma
MESO: Mesothelioma
OV: Ovarian serous cystadenocarcinoma
PAAD: Pancreatic adenocarcinoma
PCPG: Pheochromocytoma and Paraganglioma
PRAD: Prostate adenocarcinoma
READ: Rectum adenocarcinoma
SARC: Sarcoma
SKCM: Skin Cutaneous Melanoma
STAD: Stomach adenocarcinoma
TGCT: Testicular Germ Cell Tumors
THCA: Thyroid carcinoma
THYM: Thymoma
UCEC: Uterine Corpus Endometrial Carcinoma
UCS: Uterine Carcinosarcoma
UVM: ocular melanomas
OS: Overall survival
PFI: Progression Free Interval
DSS: Disease-free survival
GO: Gene Ontology
KEGG: Kyoto Encyclopedia of Genes and Genomes
GSEA: Gene set enrichment analysis
PPI: protein-protein interaction
PI3K: Phosphatidylinositol 3-kinase
AKT: protein kinase B
NOD-like receptor: Nucleotide-binding oligomerization domain-like receptors
TIDE: Tumor immune dysfunction and exclusion
ROC: Receiver Operating Characteristic
PD-1: Programmed cell death 1
PD-L1: Programmed cell death 1 ligand 1
ERAD: endoplasmic reticulum-associated degradation
GOLT1B: Golgi transport protein 1B
ATF6: the activation of transcription factor 6
STT3B: Oligosaccharyltransferase subunit
ΔNp63α: Delta - N p63 alphaGRP78: Glucose - regulated Protein 78
FOXO3a: Oligosaccharyltransferase subunit
GDSC: Genomics of Drug Sensitivity in Cancer
FLT3: FMS - like tyrosine kinase 3
ITD: Internal Tandem Duplication
CTLA-4: Cytotoxic T-lymphocyte-associated protein 4.
