# Supplementary material for: Pan-cancer analysis of TMED2: unraveling potential immune characteristics and prognostic value in cancer therapy
Source: Front Immunol. 2025 May 30;16:1578627. doi: 10.3389/fimmu.2025.1578627 (PMC12162309; doi:10.3389/fimmu.2025.1578627)
Supplement: Supplementary file 1 [file DataSheet1.pdf]

EXtended Data in Fig.7A: The original Western blot of U87 for multiple repeats.

a: U87shControl

b: U87shTMED2

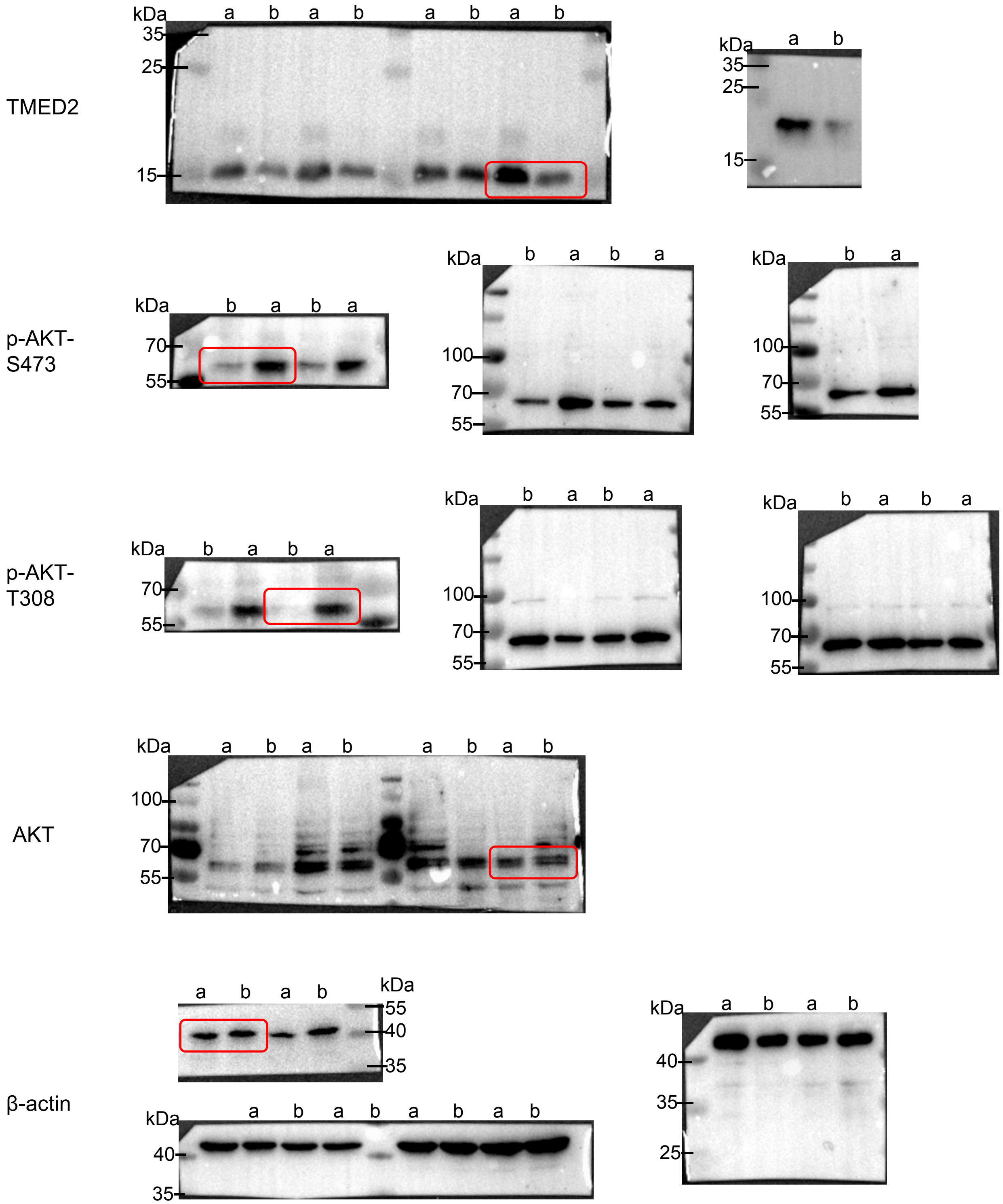

Protein markers：Thermo Scientific™，#26616

EXtended Data in Fig.7A: The original Western blot of U251 for multiple repeats.

a: U251shControl

b: U251shTMED2

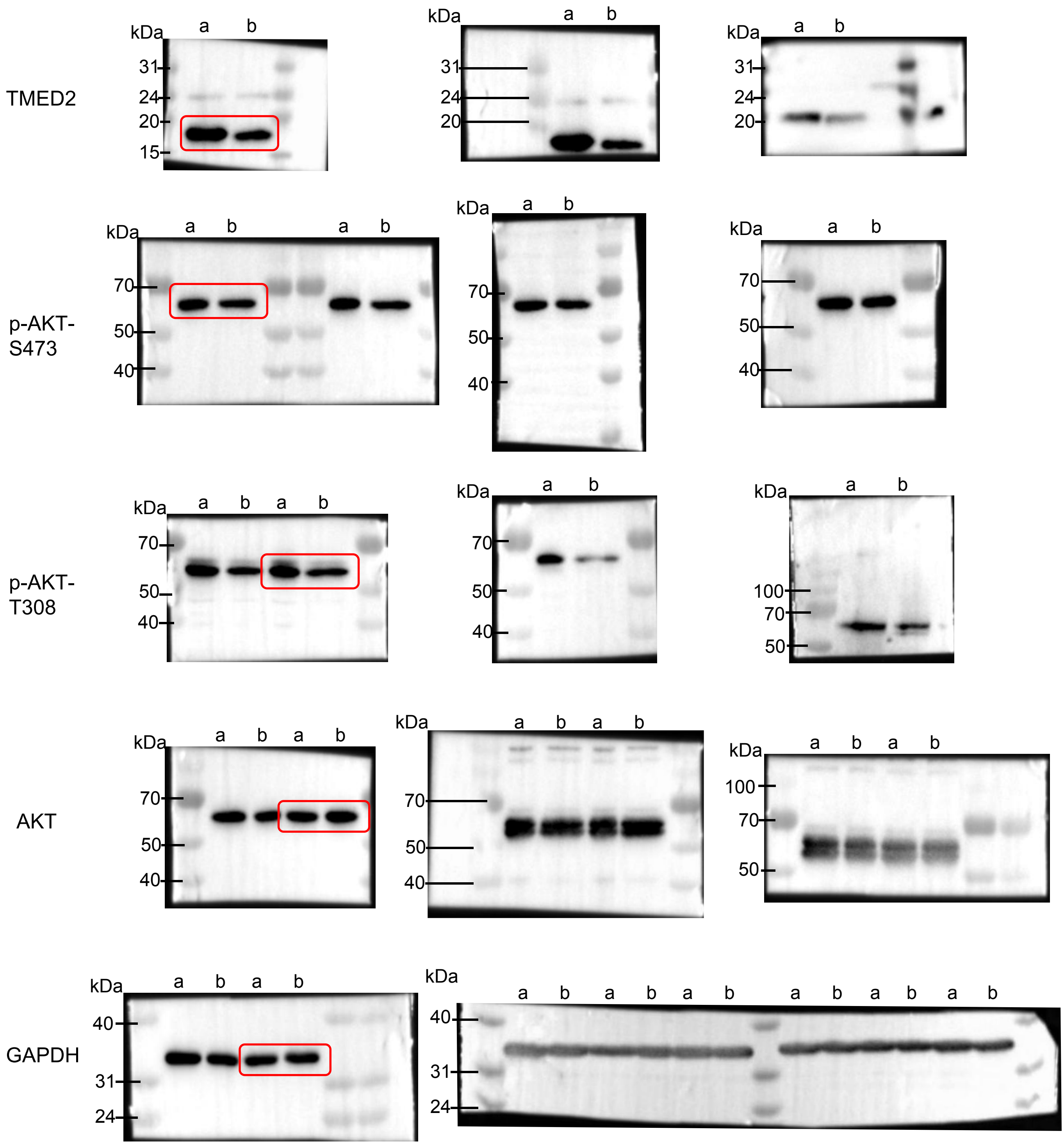

Protein markers： SparkJade， #EC1019
