# Supplementary material for: Pan-cancer analysis of TMED2: unraveling potential immune characteristics and prognostic value in cancer therapy
Source: Front Immunol. 2025 May 30;16:1578627. doi: 10.3389/fimmu.2025.1578627 (PMC12162309; doi:10.3389/fimmu.2025.1578627)
Supplement: Supplementary file 3 [file DataSheet3.pdf]

Supplementary Materials

Table. S1. Antibodys for Western blotting

Antibody	Source	RRID
Anti-TMED2	Proteintech, #11981-1-AP	AB_2204353
Anti-GAPDH	Cell Signaling Technology, #2118	AB_561053
Anti-β-actin	Cell Signaling Technology, #4970	AB_2223172
Anti-AKT	Cell Signaling Technology, #2920	AB_1147620
Anti-p-AKT S473	Cell Signaling Technology, #4060	AB_2315049
Anti-p-AKT T308	Cell Signaling Technology, #13038	AB_2629447
Peroxidase AffiniPure Goat Anti-Mouse IgG (H&L)	Jackson, #115–035-003	AB_10015289
Goat anti-Rabbit IgG (H&L) HRP conjugate	Merck Millipore, #AP156P	AB_91699

Table. S2. Primers for RT-qPCR

Gene	Primer
TMED2 F	GCTCCAAAAGGACAAGATATGG
TMED2 R	TGTCGTTGATGGCTCTGTG
β-actin F	ACCTTCTACAATGAGCTGCG
β-actin R	CCTGGATAGCAACGTACATGG

Table. S3. Gene markers for distinguishing different cell types

Cells	Markers
Cancer cells	CDH1, EPCAM, CDKN2A
Endothelial cells	EGFL7, EMCN, PECAM1
Lymphocytes	CD28A, CD27, PRF1
Macrophages	CD163, FCGR2A
Smooth muscle cells	ACTG2
Endometrial stromal cells	SUSD2
Fibroblasts	COL1A2, APOD
B cells	CD19, CD79A
T cells	CD3D, CD3E

Fig. S1. Paired expression profile of TMED2 and its expression in individual cancer staging and tumor histology, related to Figure 1.

(A) TMED2 expression in the paired normal and tumor samples analyzed by TCGA dataset.

(B-F) Expression levels of TMED2 in the clinical staging of CESC (B), the pathological tumor staging of MESO (C), the histological grading of LGG (D), the histological classification of CESC (E), and the histological classification of UVM (F).

*$P < 0.05$, **$P < 0.01$, ***$P < 0.001$, ns, not significant

Fig. S2. The Survival analysis of TMED2 on OS by the KM analysis, related to Figure 1: KICH (A), KIRC (B), KIRP (C), LUAD (D), SARC (E), THCA (F).

*$P < 0.05$, **$P < 0.01$, ***$P < 0.001$, ns, not significant

Fig. S3. PFI and DSS analysis of TMED2, related to Figure 1.

(A) Forest plot of survival analysis of TMED2 in PFI.

(B-G) The Survival analysis of TMED2 on PFI by the KM analysis: ACC (B), CESC (C), LGG (D), MESO (E), OV (F), UVM (G).

(H) Forest plot of survival analysis of TMED2 in DSS.

*$P < 0.05$, **$P < 0.01$, ***$P < 0.001$, ns, not significant

Fig. S4. The correlation between TMED2 expression and gene mutation status, related to Figure 2: BLCA (A), CESC (B), KIRC (C), KIRP (D), LUAD (E), LUSC (F), SARC (G), OV (H).

*$P < 0.05$, **$P < 0.01$, ***$P < 0.001$, ns, not significant

Fig. S5. Relationship of TMED2 expression level with immune cells infiltration in tumor microenvironment, related to Figure 3.

(A-D) The relationship between TMED2 expression and immune cells infiltration using CIBERSORT (A), MCPcounter (B), Quantiseq (C) and TIMER (D) analysis.

*$P < 0.05$, **$P < 0.01$, ***$P < 0.001$, ns, not significant

Figure S6. PPI network diagram of TMED2 and 299 co-expressed genes, related to Figure 6.

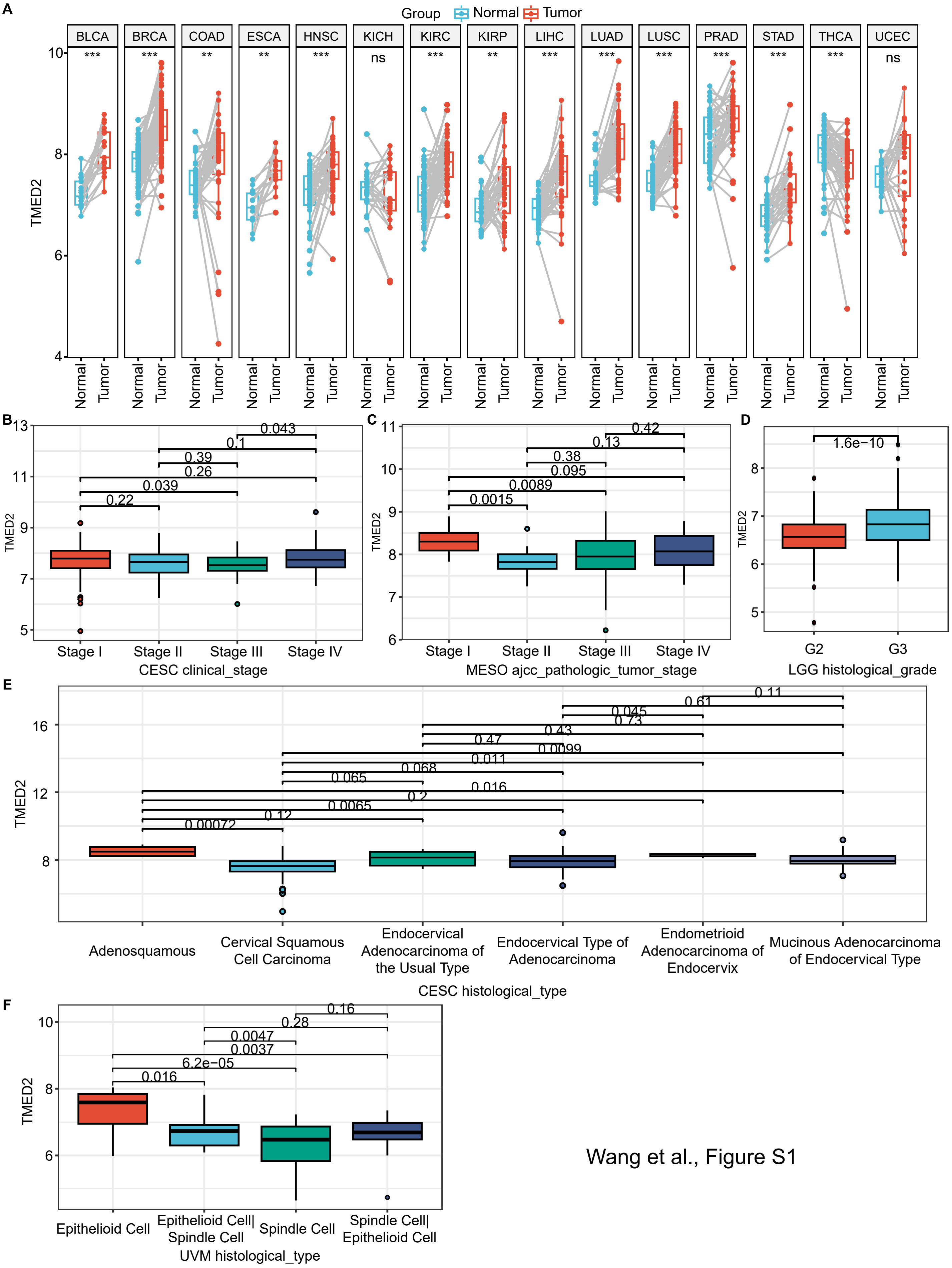

Wang et al., Figure S1

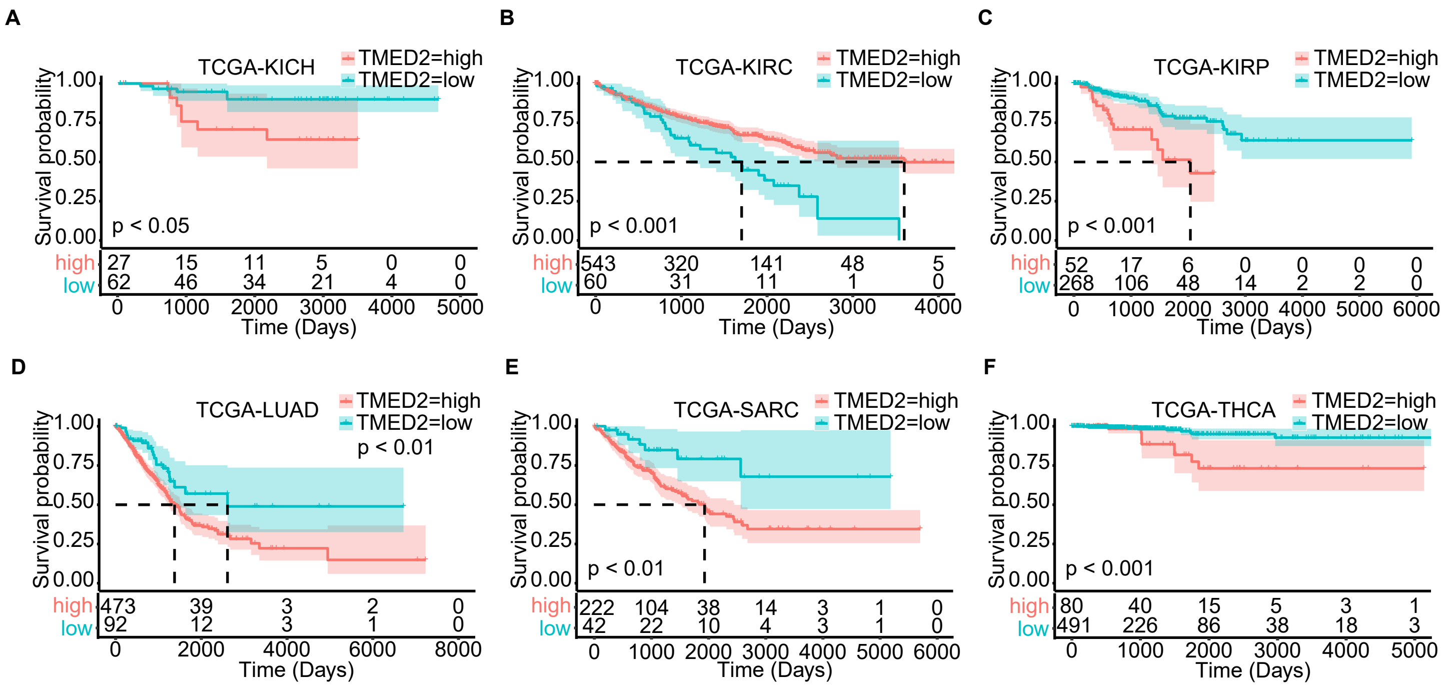

Wang et al., Figure S2

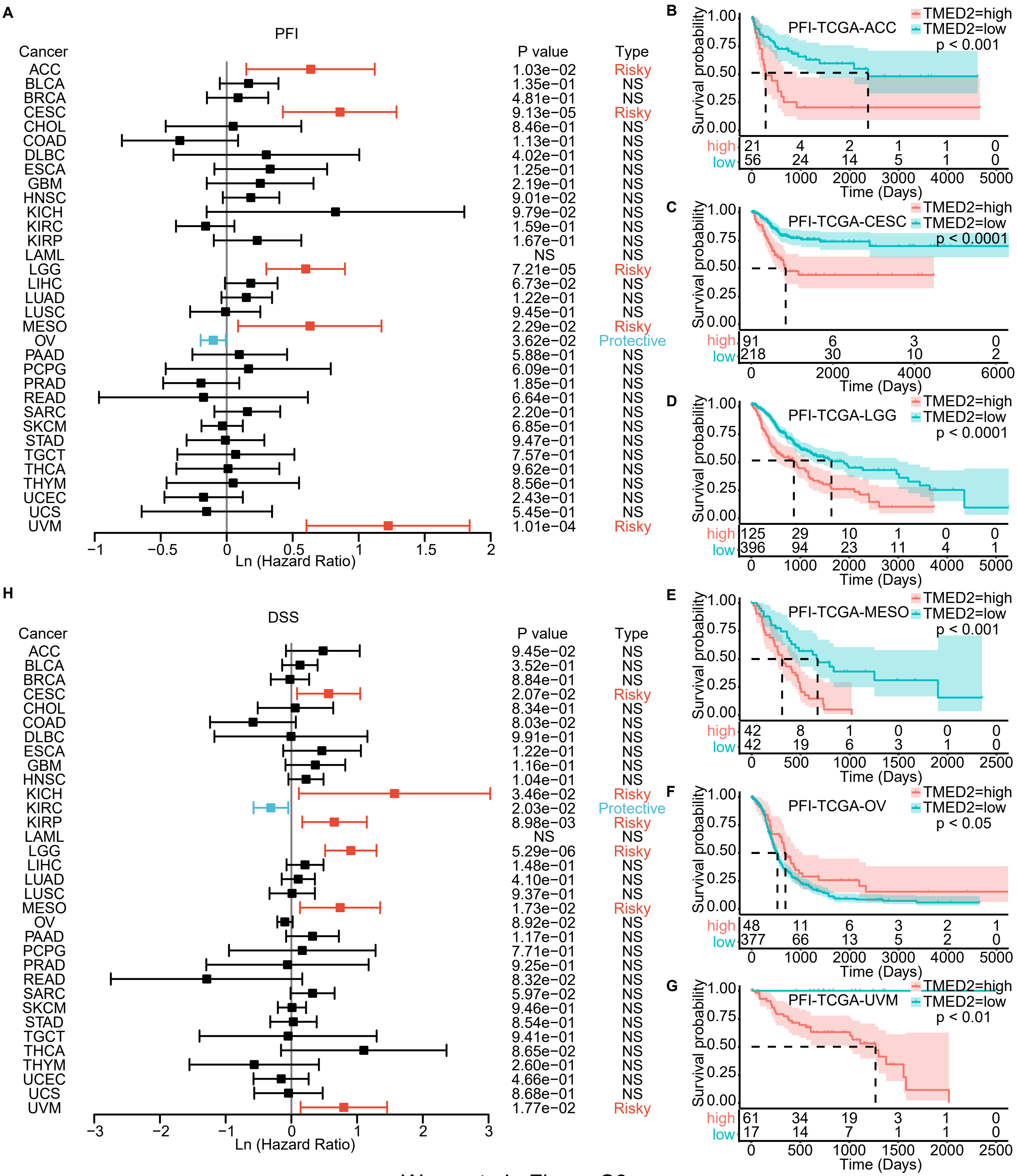

Wang et al., Figure S3

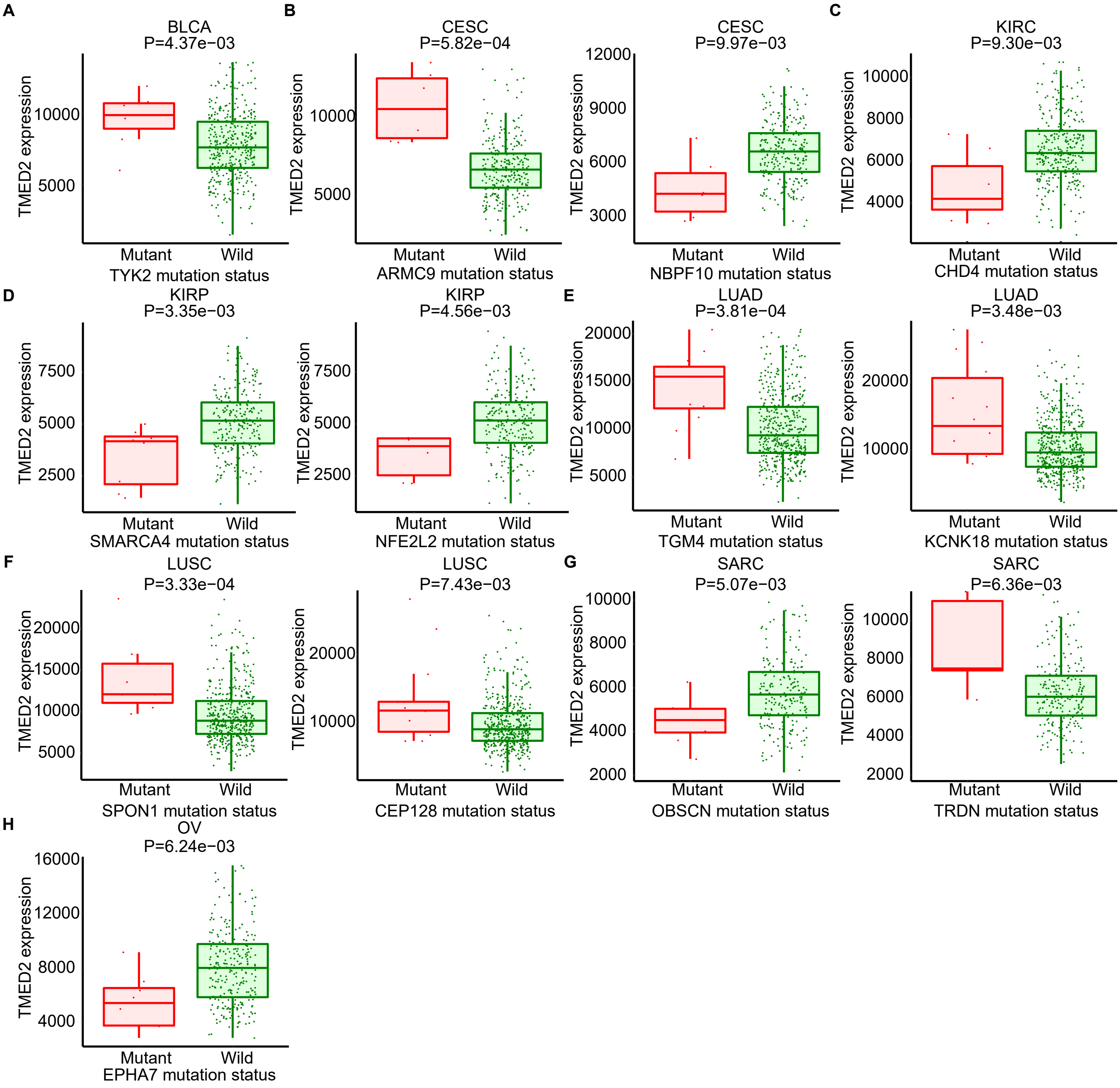

Wang et al., Figure S4

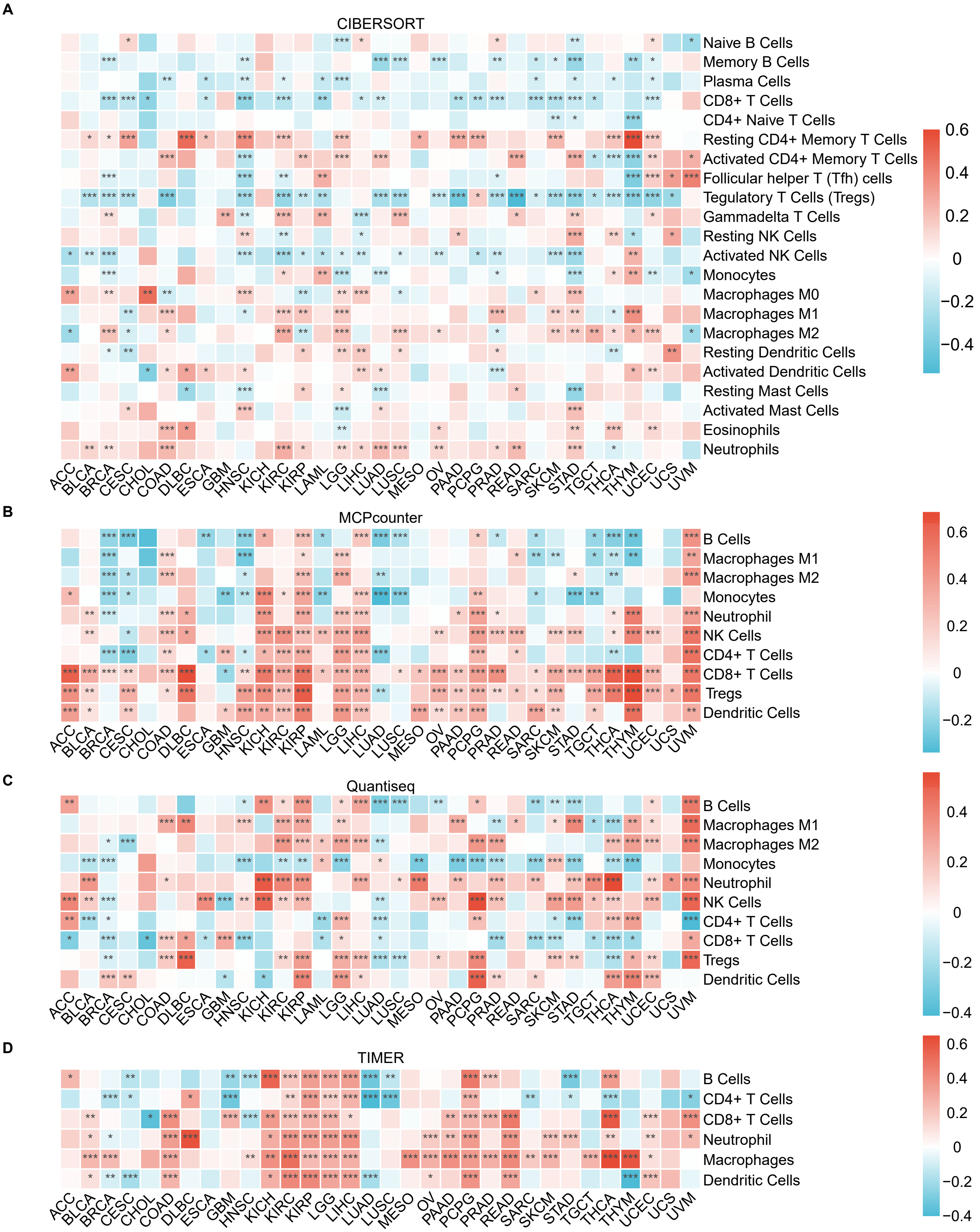

Wang et al., Figure S5

A

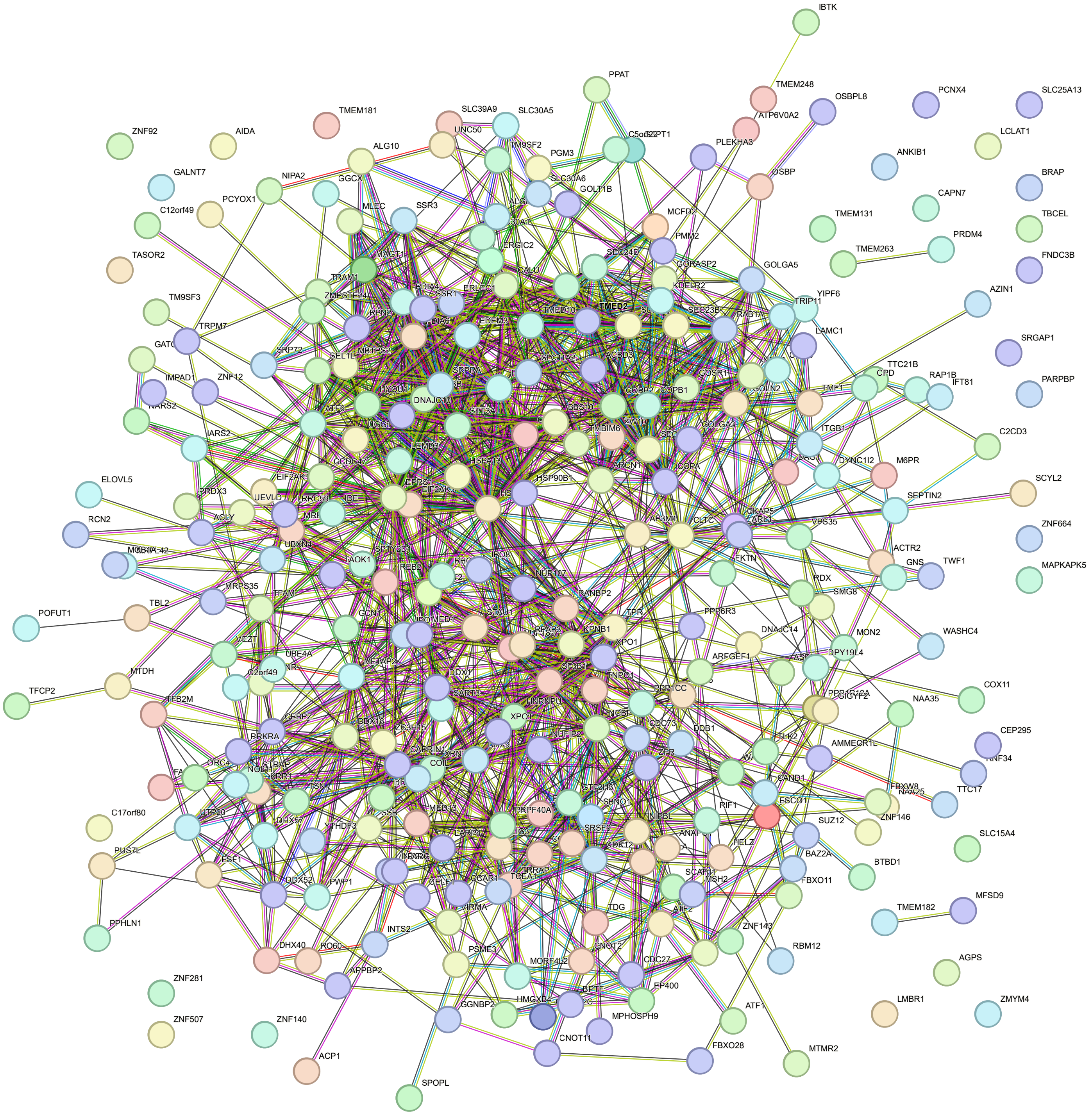

Wang et al., Figure S6
